# Changes in Social Engagement Patterns Among People With Mild Cognitive Impairment

**DOI:** 10.1093/geroni/igaf122.566

**Published:** 2025-12-31

**Authors:** Takashi Amano

**Affiliations:** Rutgers University Newark, Newark, New Jersey, United States

## Abstract

Promoting engagement in social activities may be an effective intervention to maintain or improve well-being in later life. People with mild cognitive impairment may be particularly vulnerable to disengagement from social activities. Nevertheless, little is known about social engagement among individuals with this condition, leading to a lack of practical interventions. This study aims to examine how patterns of social engagement change over time among individuals with mild cognitive impairment. Data were drawn from the Health and Retirement Study (HRS). A pooled sample was created using pairs of baseline and follow-up waves (2012–2016, 2014–2018). The final sample comprised 1,112 individuals with Cognitive Impairment No Dementia (CIND). Eight indicators were utilized to measure social engagement, including engaging in activities with grandchildren, volunteering, charity work, attending educational courses, participating in organizations, meeting up with others, having conversations, and writing or emailing. Latent transition analysis was conducted to identify transitions in patterns of social engagement. Three distinct patterns were identified at each wave: low engagement (30.6%), informal social engagement only (51.0%), and both formal and informal social engagement (18.4%). The vast majority of participants (91.3%) maintained their social engagement patterns over four years. These findings suggest that individuals with CIND can remain socially engaged over time. However, those with low engagement levels may continue to be disengaged in the absence of targeted interventions. Future research is needed to identify factors that contribute to changes in social engagement, including individual characteristics, social support networks, and external influences such as community programs.

